# Clinical and preclinical insights into a novel *MDM2::PDGFRA* fusion in recurrent glioblastoma

**DOI:** 10.1038/s41698-025-01076-4

**Published:** 2025-08-16

**Authors:** Catherine Z. Beach, Christopher A. Febres-Aldana, Juan Luis Gomez Marti, Saeed Asiry, Tamika Wong, John A. Boockvar, Randy S. D’Amico, Romel Somwar, Monika A. Davare, Morana Vojnic

**Affiliations:** 1 Department of Pediatrics, Oregon Health Sciences University, Portland, OR USA; 2 Laboratory of Pathology, National Cancer Institute, National Institute of Health, Bethesda, MD USA; 3 Department of Pathology, Lenox Hill Hospital, New York, NY USA; 4 Department of Neurological Surgery, Lenox Hill Hospital, Northwell, New York, NY USA; 5 Department of Pathology and Laboratory Medicine, Memorial Sloan Kettering Cancer Center, New York, NY USA; 6 Human Oncology and Pathogenesis Program, Memorial Sloan Kettering Cancer Center, New York, NY USA; 7 Rutgers Cancer Institute, New Brunswick, NJ USA

**Keywords:** CNS cancer, CNS cancer

## Abstract

Glioblastoma is an aggressive and treatment-refractory primary brain tumor with limited therapeutic options and high recurrence. The molecular heterogeneity of glioblastoma poses a significant challenge to therapeutic development, as targeted therapies have mostly failed in small-scale clinical trials, underscoring the need for comprehensive next-generation sequencing (NGS) characterization to identify mechanisms of resistance. In this study, we identify and functionally characterize a novel amplified fusion, *MDM2* (exon 1)::*PDGFRA* (exon 8), mediating resistance to cetuximab in an *EGFR*-amplified glioblastoma. The fusion results in a truncated PDGFRA isoform, in vitro assays demonstrate that *MDM2::PDGFRA* acts as a constitutively active oncogenic driver with a distinct sensitivity profile to tyrosine kinase inhibitors. Analysis of a glioblastoma cohort indicates *PDGFRA* structural variants often co-occur with amplification and may serve as biomarkers. These findings highlight the importance of repeat NGS profiling in clinical management and provide a translational framework for identifying and targeting emergent fusion-driven alterations.

## Introduction

Glioblastoma (GBM) is an aggressive malignancy of the central nervous system (CNS), which is characterized by high recurrence rates and dismal survival outcomes. Despite aggressive multimodal treatment—including maximal surgical resection, adjuvant temozolomide (TMZ) chemotherapy, and radiation therapy (RT)—the median survival remains 14–16 months with a five-year survival rate below 5%^1,2^. The refractory nature of glioblastoma to conventional therapies can be traced to extensive inter- and intra-tumoral heterogeneity, driven by diverse oncogenic mutations and complex transcriptional dysregulation, making it a uniformly fatal disease^3–8^.

The diagnosis of glioblastoma was historically defined by histopathological features such as vascular proliferation, pseudopalisading necrosis, and invasive growth into adjacent brain tissue^9^. The fifth edition of the WHO Classification of Tumors of the Central Nervous System (WHO CNS) was released in 2021^10^, which now incorporates molecular markers into the diagnostic criteria, highlighting a shift towards an individualized multidimensional approach to diagnosis and treatment design^11^. Indeed, molecular profiling has identified biomarkers such as mutations in isocitrate dehydrogenase (*IDH*), the promoter of telomerase reverse transcriptase (*TERT*), and *BRAF*, and *EGFR* amplifications. These somatic alterations are now instrumental in stratifying glioblastoma subtypes, predicting patient outcomes, and informing therapeutic strategies^12–14^.

Tyrosine kinase gene fusions are a prime example of actionable events that are increasingly identified due to the implementation of molecular profiling using both DNA and RNA in routine clinical management^15^. Nearly 30-50% of glioblastoma patients are predicted to harbor a gene fusion^16^. Depending on the kinase genes involved and the resulting protein structure, gene fusions can significantly affect cellular signaling, growth, and survival pathways, making them critical contributors to oncogenesis. In other solid tumors, particularly non-small cell lung cancer (NSCLC), kinase gene fusions have dramatically transformed the therapeutic landscape, as the development of tyrosine kinase inhibitors (TKI) has significantly improved patient outcomes^17–19^.

In contrast to NSCLC—where receptor tyrosine kinase (RTK) fusions such as *ALK*, *ROS1*, and *RET* are critical biomarkers predicting durable responses to tyrosine kinase inhibitors (TKIs)—the clinical efficacy of TKIs in glioblastoma remains limited. This discrepancy reflects several challenges posed by glioma biology and the CNS environment: limited CNS penetration of many tyrosine kinase inhibitors (TKIs) due to blood–brain barrier exclusion; absence of a recurrent, prevalent, and actionable oncogenic driver; and rapid therapeutic resistance mediated by redundant signaling networks^20–22^. Additionally, kinase fusions in glioblastoma frequently occur as subclonal events within extrachromosomal DNA amplifications^6–8^, unlike the lineage-defining, clonally-dominant fusions observed in NSCLC^23^.

This report presents the discovery and molecular characterization of a novel *MDM2::PDGFRA* gene fusion identified in a patient with recurrent glioblastoma. We describe the patient’s clinical trajectory, including therapeutic interventions and disease progression, and provide functional validation of *MDM2::PDGFRA*’s oncogenic potential and sensitivity to TKIs. These findings enhance our understanding of glioblastoma-associated genetic alterations and underscore the significance of identifying and functionally characterizing novel gene fusions as potential biomarkers and therapeutic targets in glioblastoma.

## Results

### Clinical presentation

**Fig. 1:**
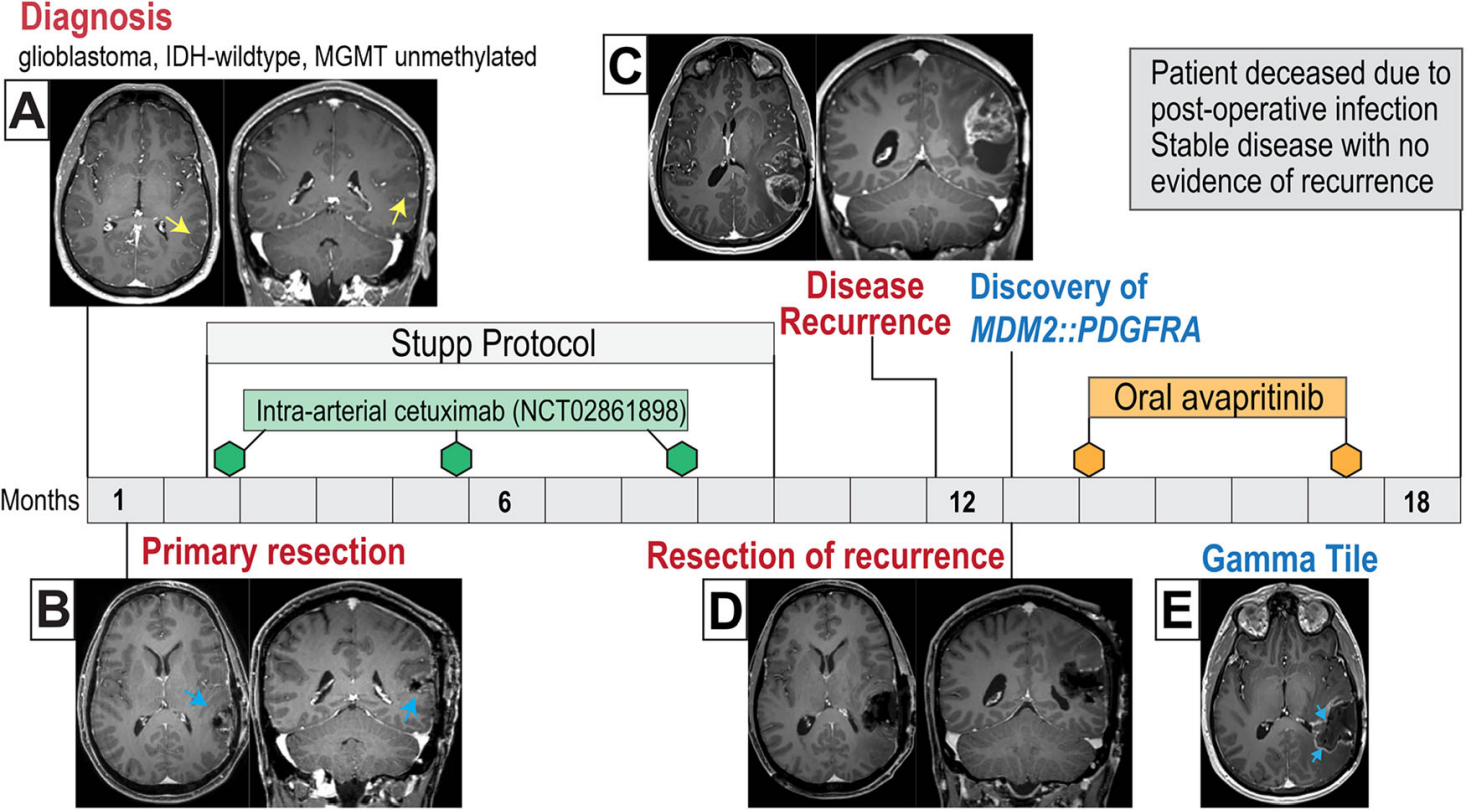
Patient’s clinical course with radiographic findings. **A** Initial presentation, axial and coronal T1 MRI images demonstrate T2 expansile lesion in the left posterior superior temporal gyrus with small area of enhancement near the superior temporal sulcus. **B** Post-operative axial and coronal T1 scan following initial resection demonstrating gross total resection. **C** Post-gadolinium axial and coronal T1 images reveal disease progression with a new cystic mass with peripheral enhancement in the left temporal lobe. **D** Post-operative axial and coronal T1 scan following second resection demonstrating gross total resection. **E** Axial T1 images demonstrate correct intraoperative placement of GammaTile brachytherapy.

A 38-year-old male presented with new-onset seizures. Initial MRI revealed a T2-weighted expansile lesion in the left posterior superior temporal gyrus with a small area of enhancement near the superior temporal sulcus **(**Fig. 1A**)**. Two weeks after presentation, the patient underwent a gross total resection **(**Fig. 1B**)**, and histopathology established a diagnosis of glioblastoma. Molecular characterization confirmed *IDH1* and *IDH2* wild-type status by PCR and an unmethylated *MGMT* promoter via pyrosequencing. Comprehensive molecular profiling using targeted NGS (DNA sequencing and RNA sequencing) revealed oncogene amplifications of *EGFR* (23 copies), *CDK4* (26 copies), and *MDM2* (171 copies) and was negative for fusions or oncogenic isoforms. Pathogenic variants included a *TERT* promoter mutation (c.228 C > T, variant allele frequency [VAF] 46%) and a *TP53* mutation (c.488 A > G, p.Y163C, VAF 47%). The tumor exhibited a low tumor mutation burden (TMB) and microsatellite stability. Given the identified *EGFR* amplification, the patient was enrolled in clinical trial NCT02861898, an open-label Phase I/II study investigating the efficacy of cetuximab, a monoclonal antibody targeting EGFR. The trial protocol includes three doses of intra-arterial cetuximab administered on post-surgery days 30, 120, and 210, in combination with standard radiation therapy and temozolomide chemotherapy, followed by maintenance temozolomide as defined by the Stupp protocol^24,25^.

Five months after treatment completion, the patient presented with worsening headaches and MRI imaging revealed a large cystic mass with peripheral enhancement in the left temporal lobe, concerning for disease recurrence **(**Fig. 1C**)**. One week later, the patient underwent a second craniotomy for maximal safe resection of the recurrent tumor. Intraoperatively, GammaTile® brachytherapy was utilized^26^, which involves placing collagen tiles embedded with Cesium-131 seeds along the resection cavity to provide localized radiation therapy. Postoperative imaging confirmed gross total resection and correct placement of GammaTile® seeds **(**Fig. 1D, E**)**.

### Detection of *MDM2::PDGFRA* fusion in recurrent glioblastoma

**Fig. 2:**
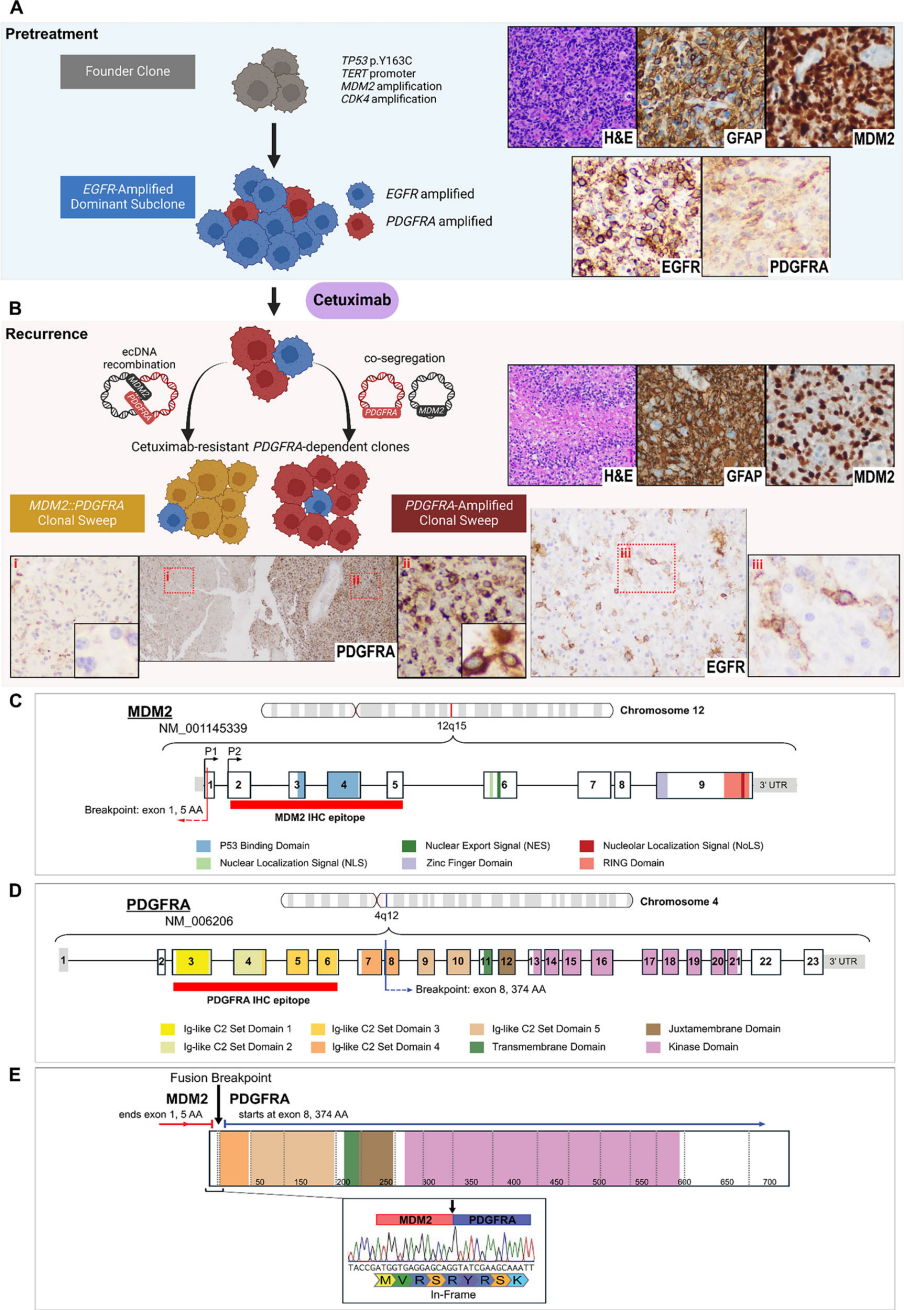
Detection of *MDM2::PDGFRA* as a mechanism of resistance to cetuximab in glioblastoma. **A** Molecular pathology of glioblastoma before treatment. H&E reveals a malignant glial neoplasm characterized by hypercellularity, marked nuclear atypia, brisk mitotic activity, microvascular proliferation, and GFAP positivity. *TP53*, *TERT* promoter, *MDM2*, and *CDK4* alterations were detected in all samples and can be regarded as founder events. Amplification of *EGFR*, but not of *PDFGRA*, was detected on initial sequencing. By IHC, EGFR showed dominant overexpression (intense in ~90%); however, a small subset was PDGFRA-positive (moderate-to-intense in ~5%), suggestive of a minor *PDGFRA*-amplified subclone undetected by bulk sequencing. **B** Following treatment with cetuximab, *MDM2::PDGFRA* and *PDGFRA* amplification were detected, and *EGFR* amplification disappeared, indicating that a major clonal sweep developed in the recurrent tumor. PDGFRA IHC was heterogeneous, ranging from negative (**i**) to strong (**ii**) immunoreactivity. By correlation of IHC Ab binding sites and fusion structure, PDGFRA-negative areas with decreased MDM2 were interpreted as MDM2::PDFGRA fusion expression, while intense PDGFRA labeling was interpreted as expression of amplified wild-type allele. EGFR IHC was markedly decreased; nonetheless, a small population retained immunoreactivity (moderate in ~10%) that may represent residual *EGFR-*amplified subclones. **C** Diagram of the genomic structure of *MDM2* (NM_001145339) located on chromosome 12q15, with exons and functional domains annotated. The fusion breakpoint, occurring in exon 1 after amino acid 5 (red line). MDM2 antibody binding epitope located between amino acids 26–169 (red band). **D** Schematic of *PDGFRA* (NM_006206) located on chromosome 4q12, showing exons and key functional domains. The fusion breakpoint, occurring in exon 8 following amino acid 374 (blue line). PDGFRA antibody binding epitope located between amino acids 32-324 (red band). **E** Diagram of the resulting *MDM2::PDGFRA* in-frame chimeric fusion gene. The translocation juxtaposes *MDM2* exon 1 to *PDGFRA* exon 8.

Pathology of the recurrent glioblastoma revealed a tumor of similar histology and immunophenotype as the pre-treatment tumor (Fig. 2A**vs. B, H&E and GFAP**). Molecular profiling (DNA sequencing and RNA sequencing) was repeated and revealed persistence of *TERT* promoter and *TP53* mutations, and *CDK4* and *MDM2* amplifications, with 19 and 121 copies, respectively. MDM2 was still overexpressed, although in some areas with a lesser proportion of tumoral cells, despite the levels of *MDM2* amplification remaining relatively unchanged (Fig. 2A**vs. B, MDM2**).

In contrast to the primary tumor, the recurrent lesion did not show *EGFR* amplification. Immunohistochemistry (IHC) showed markedly decreased EGFR expression in the recurrent tumor, post-cetuximab, compared to strong plasma membrane EGFR labeling in the pre-treatment samples (Fig. 2A**vs 2B, EGFR**). This is a significant finding given the patient’s enrollment in a clinical trial involving cetuximab and may reflect antibody-mediated clearance of *EGFR*-amplified clones.

Resistance alterations were evident in the recurrent tumor: high-level *PDGFRA* amplification (111 copies) and an in-frame *MDM2::PDGFRA* fusion – exon 1 of *MDM2* (NM_001145339.2, breakpoint 15 bp into exon 1) joined in-frame to exon 8 of *PDGFRA* (NM_006206.6) **(**Fig. 2C, D**)**. This fusion generates a truncated PDGFRA isoform with expression driven by the *MDM2* promoter, while retaining extracellular Ig-like domains 4–5 plus intact transmembrane, juxtamembrane, and kinase regions (Fig. 2E). The fusion junctional-read count (391 reads) was in the high range of NGS^27^, and concurrent *PDGFRA* amplification indicates that *MDM2::PDGFRA* itself was amplified.

We implemented PDGFRA IHC as a surrogate of *PDGFRA* alterations. PDGFRA IHC using an N-terminal antibody (aa 32-324, absent in MDM2::PDGFRA) highlighted the presence of two clones of tumor cells (Fig. 2B**, PDGFRA**): one with strong (3+) PDGFRA expression, likely derived from wild-type *PDGFRA* and a second lacking N-terminal staining – colocalizing with region of reduced MDM2 IHC (epitope aa 26-169), consistent with MDM2::PDGFRA expression. Both components were negative for EGFR. Immunoreactivity for PDGFRA was observed in a minor fraction of the pre-treatment tumor suggesting that PDGFRA-altered clones were already present (Fig. 2A, **PDGFRA**). Together, these data define a heterogeneous tumor composed of *EGFR*-amplified, *PDFGRA*-amplified, and *MDM2::PDFGRA-*harboring clones. Cetuximab drove the expansion of PDGFRA-altered clones – a treatment-resistant population (Fig. 2B).

### Landscape of *PDGFRA* structural variants in glioblastomas

**Fig. 3:**
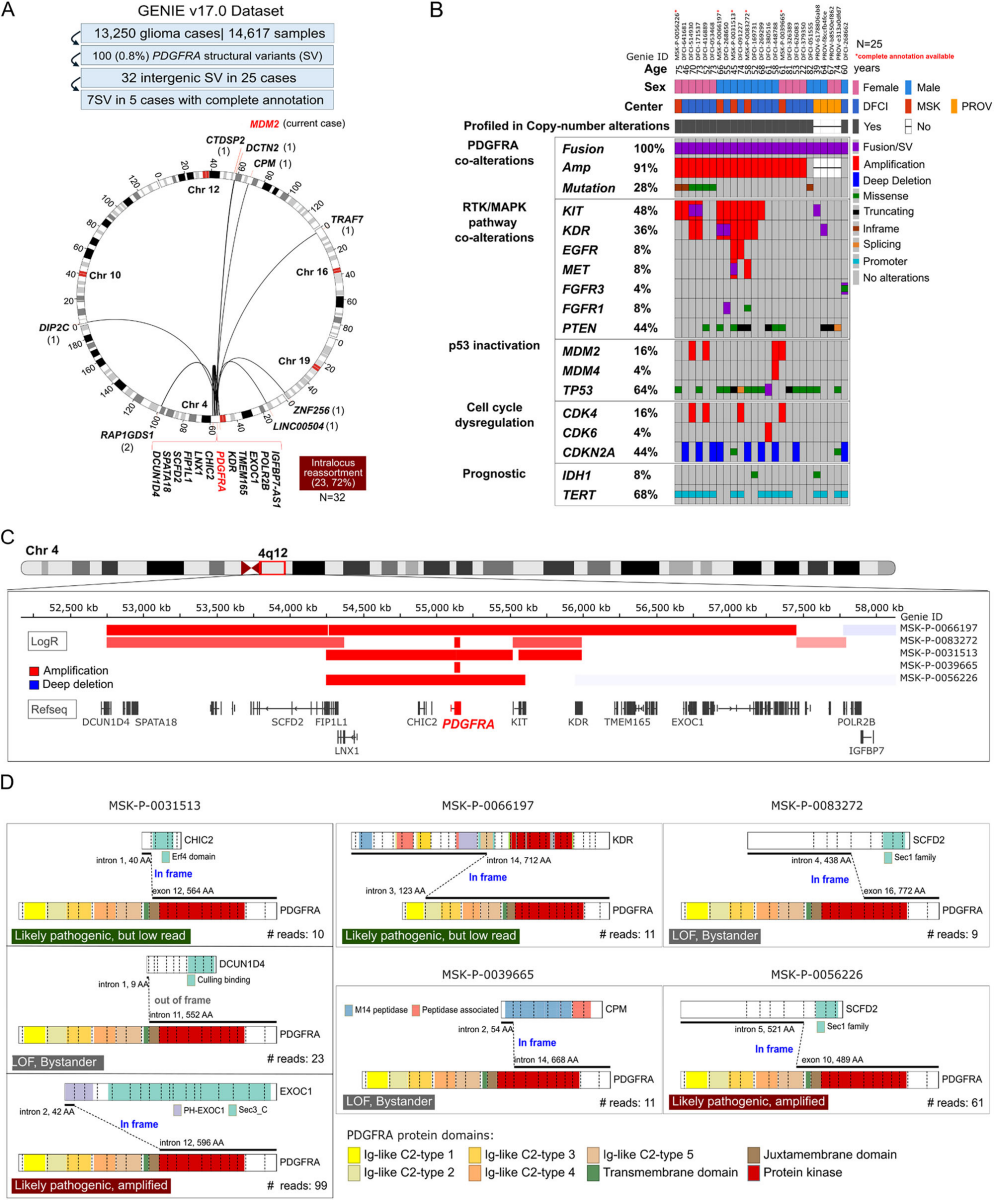
Landscape of *PDGFRA* structural variants and fusions in glioblastoma. **A** Workflow of PDGFRA structural variant (SV) selection, available in the GENIE data registry (v17.0-public), and a circos plot indicating the partner genes (bottom). A total of 32 PDGFRA SVs detected in 25 gliomas were suitable for further analysis. **B** Oncoprint showing demographics, reporting center, and genomic co-alterations from 25 gliomas (24 glioblastomas and 1 diffuse glioma) with *PDGFRA* SVs. **C** Copy number segments at the 4q12 locus for 5 cases with available copy number profiles. **D** Detailed structural variant annotation and predicted chimeric proteins of PDGFRA SVs available for 5 cases. AA: amino acid, LOF: loss-of-function (tyrosine kinase domain truncation).

The GENIE Pan-cancer cohort^28^ (v17.0-public) was examined in detail to understand the prevalence of *PDGFRA* fusions in gliomas. Out of 13,250 glioma cases available in the register, 25 (24 glioblastomas and 1 diffuse glioma) showed 32 intergenic *PDGFRA* structural variants (SVs) (Fig. 3A). The majority were the product of 4q12 intralocus reassortments (*n* = 23, 72%), and accompanied by *PDGFRA* amplification (19/21, 91%) and activating mutations (7/25, 28%). Remarkably, most samples harbored activating alterations in receptor tyrosine kinases (RTKs), including frequent co-amplifications and fusions of the *KDR* and *KIT* genes located on 4q12, as well as other mutations involving p53, cell cycle regulators, and genes frequently mutated in glioblastoma (Fig. 3B).

Complete transcript annotation, number of supporting reads, and copy number segments were available for only 5 cases with 7 SVs (Fig. 3C, D). The amplified segments were small (~36 Kb to 3 Mb) and had a high copy number (mean log2 2.9 to 4.2), which is consistent with amplifications in ecDNA^29^. SV diagrams reveal that 4 variants were predictive of a pathogenic fusion (in-frame, preserving the tyrosine kinase domain), while 3 can be considered loss-of-function/bystander effects of amplification (out-of-frame and/or TKD truncation). The number of supporting reads was interpreted as previously described^27^, and at least two *PDGFRA* fusions were amplified (MSK-P-0031513, MSK-P-0056226). Remarkably, case MSK-P-0031513 had 3 *PDGFRA* SVs involving *CHIC2*, *DCUN1D4*, and *EXOC1* (all located in 4q12), with the highest counts detected on *EXOC1::PDGFRA*, which was under stronger positive selection compared to *CHIC2::PDGFRA* and *DCUN1D4* SV.

The findings support the notion that *PDGFRA* fusions are frequently developed during the emergence of *PDGFRA* amplifications in glioblastoma.

### Clinical activity of avapritinib targeting *PDGFRA*-altered glioblastoma

Given the lack of standard-of-care treatment in recurrent glioblastoma, the patient opted for off-label use of targeted therapy after a discussion with the clinical care team. Avapritinib is a selective, type I PDGFRA-targeted TKI approved by the Food and Drug Administration^30^ for the treatment of adults with advanced gastrointestinal stromal tumors harboring *PDGFRA* exon 18 mutations, such as *PDGFRA* D842V^31^. Avapritinib demonstrates CNS penetrance in preclinical models with steady-state brain-to-plasma ratios ranging 0.74–1.00^32^, and exhibits clinical activity in PDGFRA-altered high-grade glioma patients^33^. Avapritinib treatment was initiated based on the discovery of *PDGFRA* alterations in the recurrent tumor. The patient received avapritinib for three months before succumbing to complications related to a postoperative infection after a third surgical procedure for symptomatic radiation necrosis. Examination of the tissue from this final surgery revealed no evidence of residual tumor, further suggesting that avapritinib at least successfully stabilized the tumor.

### *MDM2::PDGFRA* is a transforming oncogene with distinct TKI sensitivity

We undertook preclinical evaluation of MDM2::PDGFRA to prove its oncogenicity and sensitivity to avapritinib and other PDGFRA-targeted TKIs.

We compared MDM2::PDGFRA to the well-characterized PDGFRA D842V mutation, which served as a positive control and benchmark for evaluating tyrosine kinase inhibitor (TKI) activity. The PDGFRA D842V mutation occurs within the kinase activation loop and is known to confer resistance to type II kinase inhibitors, such as imatinib, which preferentially target kinases in the inactive conformation, characterized by an outward orientation of the conserved Aspartate-Phenylalanine-Glycine (DFG) motif (“DFG-out”)^34^. Avapritinib, a type I inhibitor, was specifically developed to target PDGFRA kinases in their active state, characterized by an inward-oriented DFG motif (“DFG-in”)^35^. The D842V mutation stabilizes the kinase in this active conformation, thereby increasing its susceptibility to type I inhibitors^31,36–38^.

To evaluate the transformative potential and inhibitor sensitivity of MDM2::PDGFRA, the Ba/F3 model system was used^39^. Derived from murine pro-B lymphocytes, Ba/F3 cells require interleukin-3 (IL-3) for growth and survival. However, expression of a transforming oncogene enables IL-3-independent proliferation, indicative of oncogenic transformation, and results in ‘onco-addiction’ to the transgene, making transformed Ba/F3 cells a robust model system for inhibitor testing^39^. *MDM2::PDGFRA* is a transformative oncogene, conferring IL-3-independent growth in Ba/F3 cells, on-par with *PDGFRA* D842V.

**Fig. 4:**
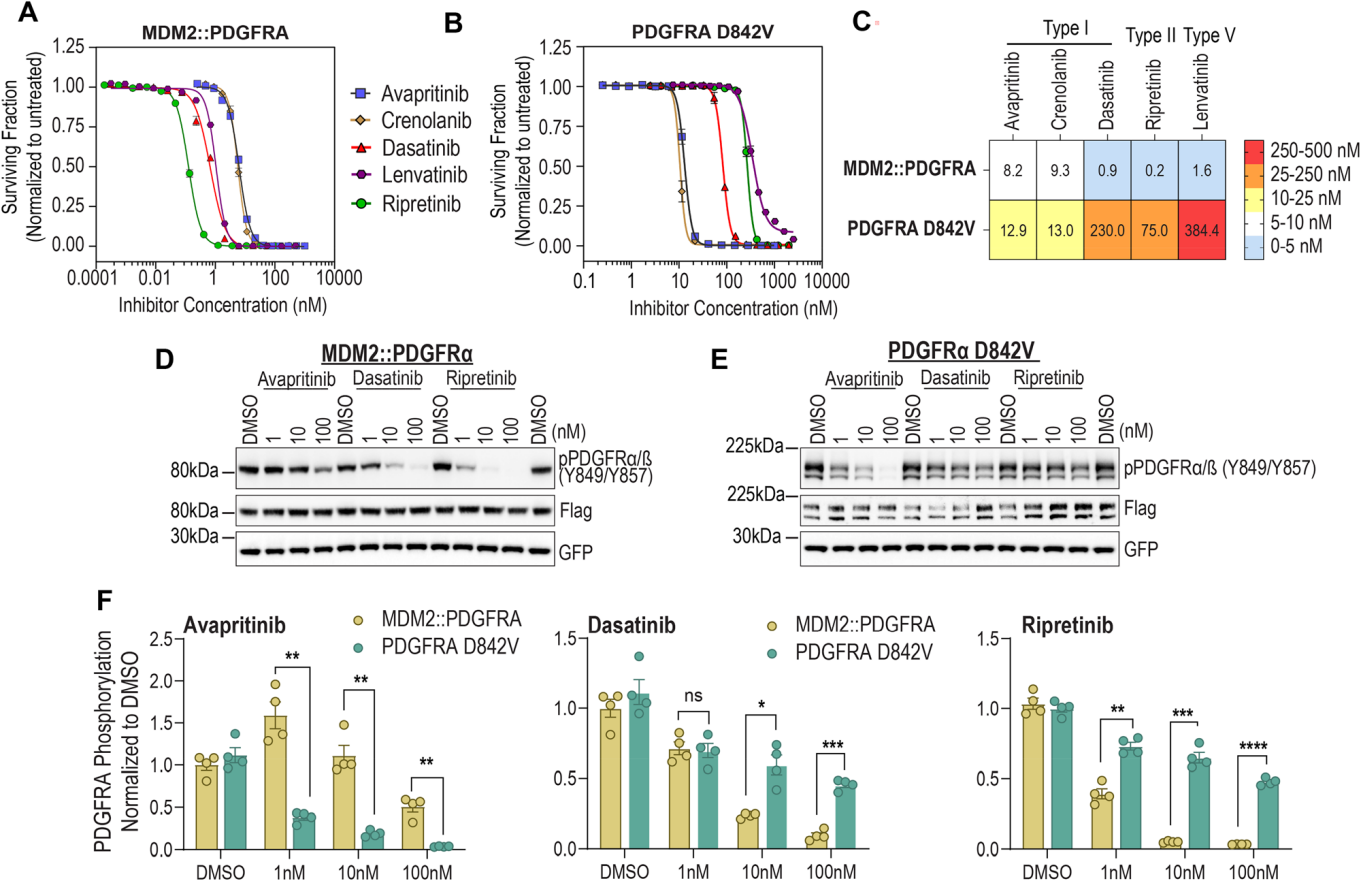
Functional characterization of MDM2::PDGFRA oncogenicity and TKI sensitivity. Dose-response cell viability comparing onco-addicted Ba/F3 cells expressing MDM2::PDGFRA (**A**) or PDGFRA D842V (positive control) (**B**) after treatment with avapritinib, crenolanib, dasatinib, lenvatinib, or ripretinib for 72 h. Representative data are average ± standard error of means (SEM) from three independent experiments, with each experiment containing three internal replicates per inhibitor. **C** Heat map summarizing inhibitory concentration (nM) required to induce 50% inhibition (IC_50_) as determined from non-linear regression analysis of dose-response assays. Average value from three replicate experiments displayed. Immunoblot analysis from Ba/F3 cells expressing MDM2::PDGFRA (**D**) or PDGFRA D842V (**E**) of phosphorylated and total PDGFRA following 2-hour treatment with DMSO (vehicle) or 1, 10, 100 nM concentrations of avapritinib, dasatinib and ripretinib. GFP, expressed from the same vector via IRES, serves as a total protein control. Data are representative of four independent experiments. **F** Densitometry graphs quantitatively compare extent of inhibition on PDGFRA after treatment with indicated inhibitors from four independent replicate experiments. Pixel density of phosphorylated PDGFRA signal was divided by total PDGFRA signal and normalized to DMSO. Significance vs. vehicle: *P ≤ 0.05, **P ≤ 0.01, ***P ≤ 0.001, ****P ≤ 0.0001.

Ba/F3 cells MDM2::PDGFRA **(**Fig. 4A**)** and PDGFRA D842V **(**Fig. 4B**)** were evaluated via dose-response cell viability assay using the following PDGFRA-targeted inhibitors: avapritinib, crenolanib, dasatinib, ripretinib, and lenvatinib. These inhibitors were selected as they exhibit distinct conformation-dependent kinase binding and inhibitor activity^40,41^. Avapritinib, crenolanib, and dasatinib are type I TKI. Ripretinib, a type II switch control inhibitor, stabilizes elements within the activation loop of the TKD into a DFG-out conformation^42,43^. Lenvatinib, a proposed Type V TKI^44^, exhibits a dual binding mode in which it occupies the ATP-binding cleft (DFG-in) while its cyclopropane moiety extends into the adjacent hydrophobic back pocket^45^, thereby combining features of both Type I and Type II inhibitors.

The cell-based 50% inhibitory concentration (IC_50_) from dose response cell viability assay revealed distinct activities of these TKI against MDM2::PDGFRA (Fig. 4A**)** versus PDGFRA D842V **(**Fig. 4B**)**. While avapritinib and crenolanib showed equivalent inhibitory activity for both PDGFRA alterations, dasatinib, ripretinib and lenvatinib were 255-, 375-, and 240-fold more potent in the MDM2::PDGFRA fusion-driven Ba/F3 cells compared to cells expressing PDGRA D842V mutant cells (Fig. 4C).

To confirm on-target activity of these inhibitors, we assessed PDGFRA autophosphorylation by immunoblotting from inhibitor-treated cell lysates. Ba/F3 cells expressing MDM2::PDGFRA **(**Fig. 4D**)** or PDGFRA D842V **(**Fig. 4E**)** were treated for 2-hours with DMSO (vehicle) avapritinib, dasatinib and ripretinib with 1, 10, 100 nM inhibitor concentrations. Densitometric analysis was performed to quantitatively assess extent of target inhibition (Fig. 4F). Comparing MDM2::PDGFRA and PDGFRA D842V: dasatinib and ripretinib significantly inhibited PDGFRA autophosphorylation of the fusion protein but not the D842V mutant (*p* < 0.05). Whereas avapritinib had modest inhibitory effect in the fusion, it was the only effective inhibitor to suppress D842V autophosphorylation. Nevertheless, cells expressing MDM2::PDGFRA were inhibited by avapritinib with IC_50_ of 8.2 nM, warranting clinical evaluation.

## Discussion

This study describes a novel, pathogenic *MDM2*(exon 1)*::PDGFRA*(exon 8) fusion detected after cetuximab treatment in an *EGFR*-amplified glioblastoma. Comprehensive genomic profiling and orthogonal IHC – including N-terminal PDGFRA and MDM2 epitope–specific antibodies – demonstrated that this fusion arose in a subset of tumoral cells. Models of co-segregation and recombination of ecDNA^6–8,29^ suggest that *MDM2::PDFGRA* could have been the result of intergenomic reassortments of *PDGFRA* and *MDM2* ecDNAs. Upon cetuximab-induced clearance of EGFR-amplified clones, subclonal populations harboring amplifications of *PDGFRA* wild type and *MDM2::PDFGRA* underwent positive selection, recapitulating the clonal dynamics of amplifications of *EGFR* wild type and EGFRvIII^7^. In vitro profiling demonstrated *MDM2::PDFGRA* yields a constitutively activated kinase sensitive to TKI. This “ecDNA-templated” RTK rearrangement effectively reprograms the tumor signaling axis at the cellular population level from EGFR to PDGFRA dependency, recapitulating features of the RTK-switch phenomena seen in other malignancies under targeted pressure (e.g., *MET* amplification after EGFR inhibition)^46–48^.

The appearance of *MDM2::PDGFRA* exemplifies glioblastoma rapid clonal evolution and “clonal sweeping,” where therapy creates a bottleneck that enriches resistant subclones^49^. Glioblastomas frequently harbor mutually exclusive RTK amplifications on ecDNA^6,50^. Particularly, treatment naïve glioblastomas with *EGFR* amplification often harbor minor *PDGFRA*-altered subclones^6–8^. Bulk NGS often misses these low-frequency drivers (<10% cancer fraction), so adopting high-resolution, multiplexed assays—such as single-cell DNA/RNA co-sequencing or spatial transcriptomics—will be critical to detect emergent fusions and inform adaptive treatment. Given the complexities of clonal evolution and the potential for resistance development, multi-target kinase inhibitors may offer a more robust therapeutic approach^51^. These inhibitors can simultaneously target multiple RTKs, potentially mitigating the impact of RTK switching and clonal selection.

While avapritinib showed clinical activity through disease stabilization, our functional studies revealed that the MDM2::PDGFRA-expressing cells were even more sensitive to non–type I inhibitors, specifically the switch-control inhibitor, type II TKI ripretinib, and the type V TKI Lenvatinib, which were 375-fold and 240-fold more potent against the fusion compared to PDGFRA D842V. Interestingly, dasatinib, a type I inhibitor, was also 255-fold more effective against MDM2::PDGFRA than D842V in cell-based assays. Dasatinib is known for broad kinase coverage and conformational adaptability^52^. Studies show structural adaptability of the dasatinib core, which, when coupled with its promiscuous nature, may explain high potency in the fusion^52^. The D842V mutant enforces a rigid DFG-in conformation, whereas the fusion harbors a wild-type but unrestrained PDGFRA kinase domain that potentially is permissive to the fusion sampling a broader range of conformations, including DFG-out states. Further, the MDM2::PDGFRA retains Ig-like domains 4 and 5 but lacks extracellular domains 1–3, which regulate autoinhibition and dimerization. The extracellular domain IgG truncation may increase conformational flexibility of the intracellular domain, enabling access to both active and inactive states. These differences may explain the varying pharmacologic properties of each inhibitor. These differences likely reflect the altered architecture of the fusion protein. MDM2::PDGFRA retains PDGFRA Ig-like domains 4 and 5, the transmembrane segment, juxtamembrane, and kinase domains, but lacks Ig-like domains 1–3. These N-terminal domains contribute to ligand-induced dimerization and autoinhibition. Their loss likely increases conformational malleability of the intracellular domain, enabling sampling of both DFG-in and DFG-out conformations. Importantly, as highlighted by Klug et al., the Type I/II/V classification system is a simplification that does not reliably predict inhibitor behavior in fusion proteins or rearranged kinases, where domain loss alters regulatory control^53^. Inhibitor binding is often context-dependent, influenced by protein flexibility, cellular environment, and allosteric modulation, factors that are not captured by static structural definitions.

These preclinical findings underscore the importance of context-dependent therapeutic strategies for *PDGFRA*-altered glioblastomas. While avapritinib was clinically effective in this patient, the broader sensitivity of MDM2::PDGFRA to type II and type V inhibitors suggests that alternative PDGFRA-targeted TKIs could have greater efficacy in *PDGFRA* fusion-driven tumors with similar structural topology. However, CNS penetration remains a critical determinant of clinical utility in brain tumor treatment, as a TKI with only moderate in vitro activity but high CNS bioavailability may ultimately be more effective than a potent inhibitor with poor CNS penetration, a point illustrated by previous failed clinical trial experience with lenvatinib and dasatinib in the treatment of recurrent glioblastoma^54,55^.

Future in vivo studies are necessary to evaluate the pharmacokinetics, blood-brain barrier permeability, and comparative therapeutic efficacy of type I and type II PDGFRA TKI inhibitors in glioblastoma models with *PDGFRA* alterations.

## Methods

### Generation of cDNA constructs

*PDGFRA* in pcDNA3.1 was purchased from the DNASU plasmid repository. The full-length PDGFRA cDNA was cloned using the EcoRI and XhoI sites in the multiple cloning site of pENTR4-No ccDB (696-1) vector (Addgene Plasmid #17424) via In-Fusion™ cloning using PCR amplification that included the addition of a C-terminal Flag tag. Simultaneously, pCX4-puro was converted into a Gateway™ Destination vector pCX4-Puro-p2a-GFP-DEST by inserting the Gateway™ Reading Frame Cassette A (ThermoFisher) using HpaI restriction digest within the multiple cloning site. The MDM2::PDGFRA-Flag fusion was generated via In-Fusion™ cloning, and full-length PDGFRA mutants were generated using site-directed mutagenesis (QuikChange™ Mutagenesis Protocol, Agilent Technologies Inc., Santa Clara, CA, USA). Plasmids were then shuttled from the entry vector to the retroviral destination vector pCX4-Puro-p2a-GFP-DEST using the Gateway™ LR Clonase™ Enzyme mix kit (Invitrogen). Plasmid sequences were confirmed via Sanger sequencing and restriction digests.

### Cell culture

Platinum E cell lines were purchased from American Type Culture Collection (CRL-11268), and Ba/F3 cells were purchased from the Leibniz Institute DSMZ-German Collection of Microorganisms and Cell Cultures, GmbH (ACC-300). Parental Ba/F3 cells were cultured in complete medium [RPMI medium 1640 with 10% FBS, 2mmol/L L-glutamine, penicillin, streptomycin, amphotericin B, and 2 ng/mL recombinant murine IL-3]. Platinum E cells were cultured in complete medium [DMEM with 10% bovine growth serum, 2 mmol/L L-glutamine, penicillin, streptomycin, and amphotericin B]. Cell lines were maintained and passaged using standard aseptic techniques and incubated at 37 °C in a humidified atmosphere with 5% CO_2_. All cell lines were tested for mycoplasma contamination every two months using the Lonza MycoAlert^TM^ PLUS Mycoplasma Detection Kit, and antibiotics were included in all cell culture media. Cells were inspected daily for growth, morphology, and viability. Cells were passaged when they approached 75% confluency, and passage numbers were recorded to ensure consistency in experiments.

### BaF3 stable cell line generation

Platinum-E cells were transfected with pCX4-puro-p2a-GFP PDGFRA-Flag WT, D842V, or MDM2::PDGFRA-Flag using TransIT®-LT1 (Mirus Bio) DNA transfection reagent to generate replication-incompetent, ecotropic retrovirus. This virus was used to transduce BaF3 parental cells, seeded the day of transduction at 1.0 × 10^6^ cells/mL in 2 mL of media. Virus-containing media were removed after 24 h, and cells were given fresh media overnight. The next day, 48 h post-transduction, cells were selected with 2 µg/mL puromycin. Selection was completed for a minimum of 96 hours. To generate IL3-independent, stable, post-selection Ba/F3 cell lines, were washed three times with complete medium and seeded at 0.5 × 10^6^ cells per mL. Cells were counted every 2 days and were expanded as needed to maintain a density of <1.5 × 10^6^ cells per mL. Cells that grew out after IL3 withdrawal were maintained in IL3-free complete medium and used for in vitro assays. For experimental rigor, all transformed (post-IL3 withdrawal) cell lines were sequenced to verify the presence of the desired mutation. Cells were harvested, pelleted, and DNA was extracted using QuickExtract DNA Extraction Solution (Lucigen).

### Inhibitors

Crenolanib (CAS# 670220-88-9), Avapritinib (CAS# 1703793-34-3), Ripretinib (CAS# 1442472-39-0), Dasatinib (CAS# 302962-49-8), and Lenvatinib (CAS #417716-92-8) were purchased from MedChemExpress.

### Immunoblot Analysis

Stably transduced BaF3 cell lines: MDM2::PDGFRA-Flag and PDGFRA-Flag D842V were treated with avapritinib, dasatinib, or ripretinib at 1, 10, and 100 nM or DMSO as a negative control, for 2 hr at a density of 2 × 10^6^ cells per condition. Following treatment, cells were pelleted, washed once in ice-cold DPBS, and lysed in cell lysis buffer supplemented with 0.25% deoxycholate and protease and phosphatase inhibitors. Protein concentrations were determined using the Pierce^TM^ BCA Protein Assay (ThermoFisher Scientific). Protein was extracted with 4X Bolt^TM^ LDS Sample Buffer, supplemented with β-mercaptoethanol, for 10 min at 75 °C. 10 µg of extracted protein lysate was run for 1.5 hr at 144 V on a pre-cast 15-well, 4–12% Bis-Tris Gel (Invitrogen; ThermoFisher Scientific) and transferred to nitrocellulose membrane (Prometheus) under wet transfer conditions at 16 V for 18 h at 4 °C. Spectra Multicolor Broad Range Protein Ladder (Thermo Fisher Scientific) was used to determine relative molecular weights of protein bands after imaging. Four replicate sets were run for this experiment, with each set being run on two, 15-well comb gels using 10 µg total cell extracts per well. Blots were probed with antibodies specific for phospho-PDGFRA/B (Y849/Y857) (Cell Signaling Technology (CST); 3170; 1:1000), Flag (Invitrogen; 701629; 1:1000), phospho-p44/42 MAPK (pERK1/2) (CST; 4370; 1:2000), p44/42 MAPK (ERK1/2) (CST; 4696; 1:2000), phospho-S6 Ribosomal Protein (Ser235/236) (CST; 4858;1:1000), total S6 Ribosomal Protein (CST; 2317; 1:1000), GFP (Origene; TA150032; 1:5000). Western blots were imaged using the ChemiDoc™ (Bio-Rad Laboratories, Hercules, CA, USA) for detection of horseradish peroxidase-conjugated secondary antibodies or the Odyssey® DLx Imaging System (a LICOR, Lincoln, NE, USA) for detection of near-infrared fluorescent-conjugated secondary antibodies following the respective manufacturer’s protocol. Densitometry was performed using Image Lab™ 6.01 Software (RRID: SCR_014210). for blots imaged using the BioRad ChemiDoc MP imaging station and Image Studio™ Software 5.2 (LI-COR) for blots imaged using the LI-COR Odyssey imaging station.

### Cell Viability Assay

The following PDGFRA-TKI inhibitors: avapritinib, crenolanib, dasatinib, lenvatinib, and ripretinib, were prepared as 1 mM stocks diluted in DMSO and then, using the D300 Digital Dispenser (Hewlett-Packard), were distributed along a 14-point dose gradient (range: 200 pM to 1.25 µM) in 384-well plates that were pre-seeded with 25 μL of complete RPMI-10% FBS medium using the Multidrop Combi Reagent Dispenser (Thermo Scientific). Again, using the Multidrop Dispenser, BaF3 cells expressing either MDM2::PDGFRA-Flag or PDGFRA D842V were seeded into the 384-well plates prepped with inhibitors, at 1,000 cells per well in 25 µl, bringing the total volume up to 50 µl per well. Seeded plates were incubated at 37 °C in a humidified atmosphere with 5% CO_2_. for 72 hours. Cell viability was evaluated by the addition of 5 µl per well of WST-8/CCK8 tetrazolium cell reagent (Abcam) and detecting the colorimetric change at 450 nm using the BD Biotek Synergy^TM^ 2 plate reader. Absorbance data from drug-treated wells were normalized to a death and vehicle-only control in Microsoft Excel. Within each experiment, each inhibitor was tested in triplicate, and overall, each experiment was performed in quadruplicate. GraphPad Prism (RRID:SCR_002798). was used to perform non-linear regression curve fit analysis to determine 50% inhibitory concentration (IC_50_).

### Patient consent

Informed consent for publication of the case report was obtained from the patient’s next of kin. The study was approved by the Northwell Institutional Review Board (IRB) and was conducted in accordance with the principles outlined in the Declaration of Helsinki.

### Next generation sequencing

Tumor genomic profiling was performed at Caris Life Sciences using next-generation sequencing on the Illumina NovaSeq 6000 platform. The assay included targeted sequencing of clinically relevant genes alongside broader exome-level analysis. Copy number alterations, tumor mutational burden (TMB), and microsatellite instability (MSI) were assessed as part of the integrated analysis. Sequencing data were aligned to the hg38 reference genome.

### Copy number alteration

The copy number alteration (CNA) of each exon is determined by a calculation using the average sequencing depth of the sample along with the sequencing depth of each exon, and comparing this calculated result to a pre-calibrated value. If all exons within the gene of interest have an average of ≥ 3 copies and the average copy number of the entire gene is ≥ 6 copies, the gene result is reported as amplified. If an average of ≥ 4 but < 6 copies of a gene is detected, or if the average copy number of the gene is ≥ 6 copies but contains exons with an average of < 3 copies, the gene result is reported as intermediate. If the average and entire 95% confidence interval of a gene is < 1 copy, the gene result is reported as deleted. If an average > 1.9 copies but < 4 copies of a gene is detected, the gene result is reported as no amplification detected or deletion not detected.

### Gene fusion and transcript variant detection

Gene fusion and variant transcript detection were performed on mRNA isolated from a formalin-fixed paraffin-embedded tumor sample using the Agilent SureSelectXT Low Input Library prep chemistry, optimized for FFPE tissue, in conjunction with the SureSelect Human All Exon V7 bait panel (48.2 Mb) and the Illumina NextSeq 500/550 or NovaSeq 6000 sequencers. In addition to transcript variants, MI Transcriptome is designed to detect fusions occurring at known and novel breakpoints within genes. Only a portion of the genes tested are included in this report. The genes included in this report represent the subset of genes most commonly associated with cancer. All results can be provided upon request. For fusions, analytical validation of the MI Transcriptome test demonstrated ≥97% Positive Percent Agreement (PPA), ≥99% Negative Percent Agreement (NPA) and ≥99% Overall Percent Agreement (OPA) with a validated comparator method. The versioned reference identifier used for the transcript ID was Feb.2009 (GRCh37/hg19).

### MGMT methylation testing

DNA extraction from paraffin-embedded tumor samples was performed for subsequent pyrosequencer-based analysis of 5 CpG sites (CpGs 74-78). All DNA samples undergo a bisulfite treatment and are PCR amplified with primers specific for exon 1 of MGMT (GRCh37/hgl9 – chr10: 131,265,448- 131,265,560). Methylation status of PCR amplified products is determined using the PyroMark system. Samples with ≥7% and <9% methylation are considered to be equivocal or gray zone results. This assay requires samples to have at least 50% tumor nuclei.

### Immunohistochemistry

Immunohistochemistry (IHC) was performed in a CLIA-certified laboratory using formalin-fixed, paraffin-embedded tissue (tissue block submitted for NGS). GFAP (prediluted by manufacturer, clone EP672Y, Cell Marque, CA), EGFR (dilution 1:300, clone EGFR.113, Leica, IL), PDGFRA (dilution 1:200, clone OTI2E9, OriGene, MD), and MDM2 (prediluted by manufacturer, clone IF2, Cell Marque, CA) were evaluated by J.L.G.M. and S.A. with comparison of the extent and intensity of immunoreactivity of pre-treatment and post-treatment samples.

## Supplementary information

Supplementary Information

## Data Availability

The data supporting this study include clinical data that cannot be shared due to patient confidentiality and ethical restrictions. However, non-clinical datasets generated and analyzed during this study are available from the corresponding authors upon reasonable request. The data from the GENIE data registry (v17.0-public) can be accessed via: https://www.aacr.org/professionals/research/aacr-project-genie/aacr-project-genie-data/. All gliomas cases with PDGFRA structural variants were systematically reviewed and summarized according to partner genes and genomic co-alterations documented in the registry. Cases with detailed structural variant annotations, including genomic breakpoints, transcript orientation, and number of supporting reads, were analyzed as previously described^27^. Chimeric proteins were modeled using the St. Jude’s ProteinPaint tool (https://proteinpaint.stjude.org/).

## Code availability

Code sharing is not applicable to this article, as no codes were generated or analyzed during the current study.
