# Supplementary figures and images for: Clinical and preclinical insights into a novel MDM2::PDGFRA fusion in recurrent glioblastoma
Source: NPJ Precis Oncol. 2025 Aug 16;9:289. doi: 10.1038/s41698-025-01076-4 (PMC12357959; doi:10.1038/s41698-025-01076-4)

# Supplementary Information

# Figure 1D

MDM2::PDGFR $\alpha$

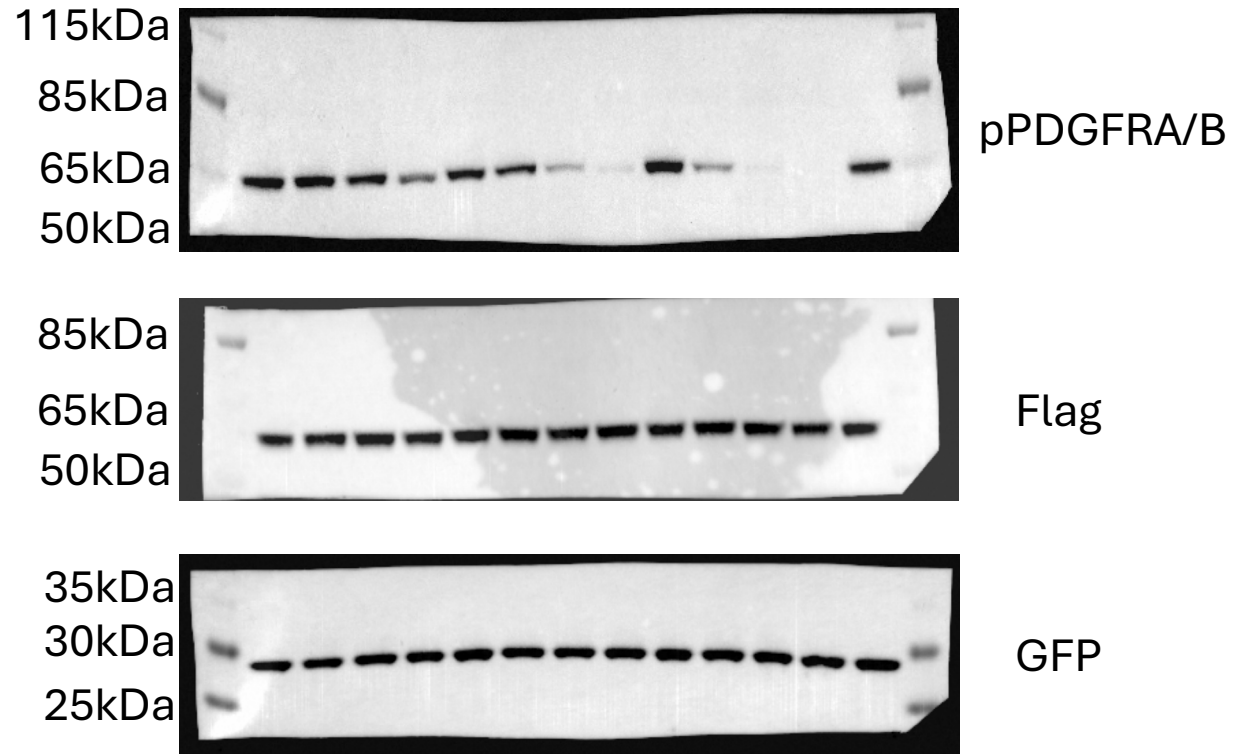

# Figure 1E

PDGFRA D842V

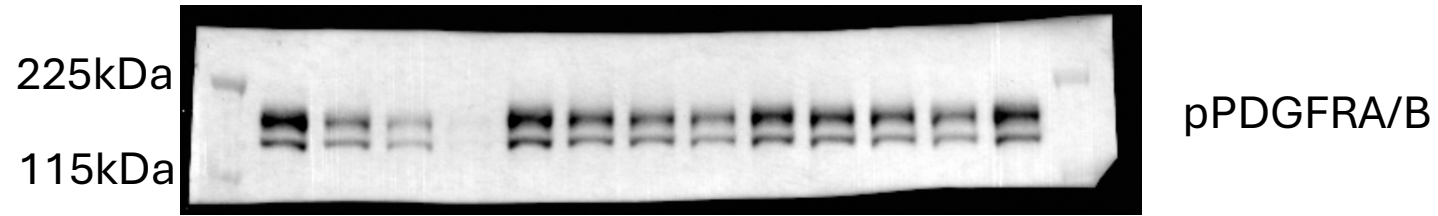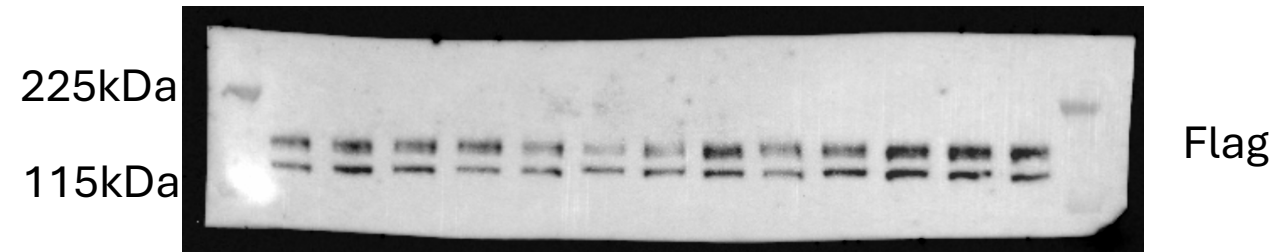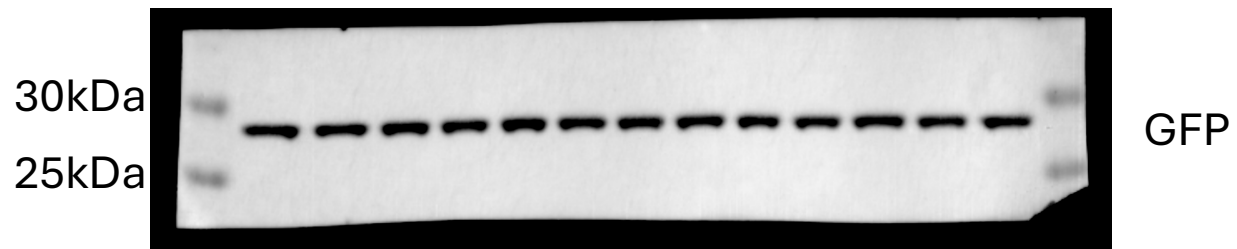

Spectra Protein Ladder

Supplement: Supplementary file 1 — Supplementary Information [file 41698_2025_1076_MOESM1_ESM.pdf]
